# Isolation of *Leptospira kirschneri* serovar Grippotyphosa from a red panda (*Ailurus fulgens*) after antimicrobial therapy: Case report

**DOI:** 10.3389/fvets.2022.1064147

**Published:** 2023-02-02

**Authors:** Karen LeCount, Kami Fox, Tammy Anderson, Darrell O. Bayles, Tod Stuber, Jessica Hicks, Linda K. Schlater, Jarlath E. Nally

**Affiliations:** ^1^National Veterinary Services Laboratories, Animal Plant and Health Inspection Service (APHIS), U.S. Department of Agriculture, Ames, IA, United States; ^2^National Center for Animal Health (NCAH) Leptospira Working Group, U.S. Department of Agriculture, Ames, IA, United States; ^3^Fort Wayne Children's Zoo, Fort Wayne, IN, United States; ^4^Infectious Bacterial Diseases Research Unit, Agricultural Research Service (ARS), U.S. Department of Agriculture, Ames, IA, United States

**Keywords:** *Leptospira*, leptospirosis, red panda, *Ailurus fulgens*, *L. kirschneri*, serovar Grippotyphosa, case report

## Abstract

A 1-year-old female red panda started showing symptoms of illness, including lethargy, anorexia, abdominal discomfort, and vomiting, shortly after transfer to a new zoo. Serum was tested for leptospirosis using the microscopic agglutination test, and a titer of 1:25,600 to serogroup Grippotyphosa was detected. Antimicrobial treatment with doxycycline was initiated. After completion of treatment and resolution of clinical symptoms, a urine sample was collected to ensure clearance of leptospires and cessation of urinary shedding prior to co-housing with other red pandas. A repeat serum sample taken 13 days later had a lower titer of 1:6,400 to serogroup Grippotyphosa. A sample of the animal's urine was cultured in HAN media and was culture positive for *Leptospira*. The recovered isolate was completely characterized by whole genome sequencing and serotyping with reference antisera, and the isolate was classified as *Leptospira kirschneri* serogroup Grippotyphosa serovar Grippotyphosa strain RedPanda1.

## Introduction

Leptospirosis is a zoonotic disease of worldwide concern. It is caused by spirochetes of the genus *Leptospira* that are typically excreted *via* urine and transmitted through contact with infected animals or their contaminated habitats (1, 2). Zoo animals are at risk of leptospirosis from interactions with wildlife reservoir hosts, such as small mammals and rodents, and from other exotic animal species (3). Employees are also at risk of infection from handling infected animals or cleaning enclosures. Several cases of leptospirosis have been documented in zoo animals (4–7).

In 2001, two eastern black rhinoceros (*Diceros bicornis michaeli)* were diagnosed with leptospirosis using the fluorescent antibody test (FAT) and the microscopic agglutination test (MAT). The disease was hypothesized to be a result of infection with *Leptospira kirschneri* serovar Grippotyphosa through wallow contamination by wild raccoons (6). Multiple animal species, including non-human primates and a desert bighorn sheep (*Ovis canadensis mexicana*), were diagnosed with leptospirosis that was associated with relocation during exhibit construction and grounds renovation in an urban zoo (5). In both cases, serovar Grippotyphosa was inferred as the cause of infection, and as the cause of death in a young adult male titi monkey (*Callicebus moloch*). A juvenile south-central black rhinoceros (*Diceros bicornis minor*) showed evidence of infection from serogroup Autumnalis. Feral trapped rats were negative for leptospirosis, but feral trapped squirrels showed evidence of exposure to both serogroup Grippotyphosa and Autumnalis (5). In 1978, two zoo workers were diagnosed with leptospirosis based on high MAT titers against serogroup Icterohaemorrhagiae and isolation of Icterohaemorrhagiae from the urine of one patient (4). Both workers had contact with bear cubs that also had titers for Icterohaemorrhagiae.

In 2022, MAT and real-time polymerase chain reaction (RT-PCR) was used to diagnose a Sumatran tiger (*Panthera tigris sumatrae*) residing in a zoo with *L. kirschneri* serovar Grippotyphosa (7). Clinical signs of illness resolved after treatment, but a urine sample collected at 595 days after initial diagnosis was PCR positive. Leptospirosis is typically treated with doxycycline; however, antimicrobial treatment or clinical resolution does not guarantee cessation of shedding. Detection of *Leptospira* after clinical resolution has been documented in both human and animal cases. A 10-year old girl shed *Leptospira* in her urine for several weeks after clinical resolution (8). A dog that was treated with intravenous penicillin, and 4 days later treated with doxycycline, continued to be positive for leptospires in its urine by dark-field microscopy 1 week later (9). In another case, *L. interrogans* serovar Canicola was isolated from a dog after both vaccination and treatment with penicillin, and in the absence of a positive titer in the MAT. This dog was not showing any acute illness but showed signs of liver dysfunction in routine bloodwork that lead to the further testing for leptospirosis (10).

The case study presented here diagnosed leptospirosis in a female red panda (*Ailurus fulgens*) while in routine quarantine after movement from another zoo. Continued evaluation post-antimicrobial treatment confirmed urinary shedding of leptospires, highlighting continued risk of transmission. Urine samples were cultured, and recovery of an isolate allowed for complete characterization by serotyping and genome sequencing.

## Case description

In mid-2019, a female *Ailurus fulgens*, commonly known as a red panda, aged 1 year, appeared clinically normal when transferred to a new zoo. On July 24, 2019, the red panda underwent a quarantine exam, routine diagnostics, and vaccinations including having blood drawn for *Leptospira* MAT and CBC/chemistries. Testing for leptospirosis, as well as other illnesses, is a part of the routine surveillance at the zoo. On exam, the panda was thin but otherwise appeared healthy. Several of the routine tests showed atypical results. Bloodwork indicated increased BUN:Creatine ratio, 43.3 ratio (US) (expected range based on best available match: 3.7–18.8). The MAT was performed at the National Veterinary Services Laboratories (NVSL) as previously described with a starting test dilution of 1:100 (11). The end point was identified as the last well with 50% or more agglutination. The sera were tested against 15 serovars (Supplementary Table 1). At that time, a titer of 1:25,600 to serogroup Grippotyphosa and lower titers to serogroups Icterohaemorrhagiae and Autumnalis (1:400 and 1:200, respectively), were detected. The high titer to Grippotyphosa was noted and additional samples were planned to be collected for follow-up testing at the next examination. Multiple fecal samples were collected since arrival at the zoo and several showed signs of cryptosporidium on ELISA testing but were negative on PCR. *Proteus mirabilis* was also present in a few of the fecal exams. Per the zoo policies, the red panda was scheduled to stay in quarantine for 30 days.

**Figure 1:**
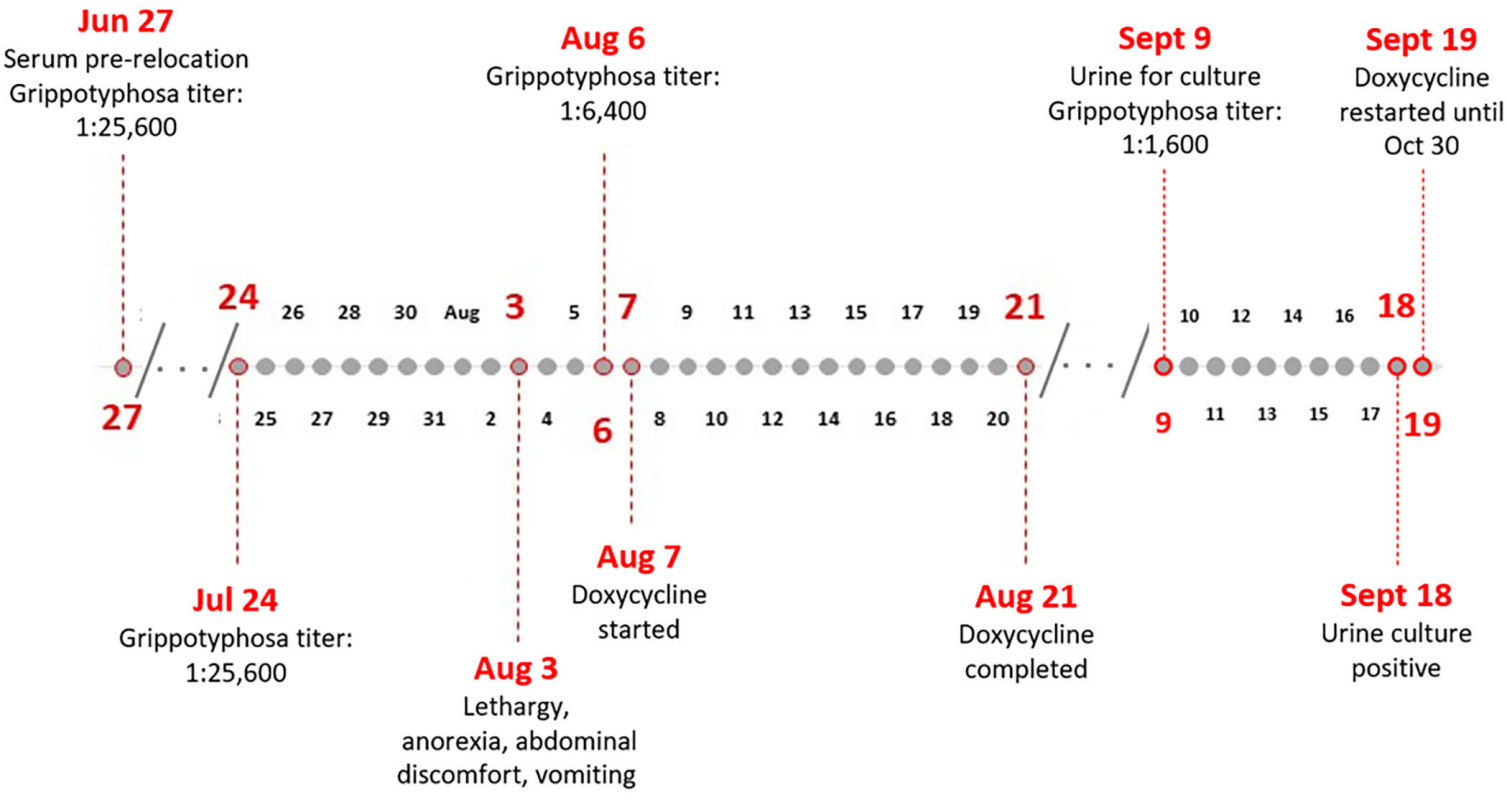
Timeline of events illustrating the red panda symptoms, treatment, and testing results.

On August 3, the red panda began to show abnormal behavior and was reported as lethargic, anorexic, with abdominal discomfort and vomiting (Figure 1). Three days later, she was examined again, and samples collected for additional testing. On this examination, she had signs of mild gastroenteritis, the likely cause of the abdominal pain and anorexia. Bloodwork showed increased basophil percentage, 7.0% (expected range: 0–4.0), neutrophil count 8.05 × 10^3^ cells/μL (expected range: 0.970–7.913), basophil count, 0.85 × 10^3^ cells/μL (expected range: 0–0.424), and total CO_2_ 27 mmol/L (expected range: 8.8–24.3) (12). Eosinophils were within normal range. The urinalysis showed a slightly elevated specific gravity at 1.042 ratio (expected range: 1.004–1.040). Serum was collected for paired serum testing in the MAT, and urine was collected by cystocentesis for RT-PCR to confirm leptospirosis. The RT-PCR was performed at an external commercial diagnostic laboratory and results were reported as negative. A repeated MAT, on August 6, showed a decrease in titer, positive at 1:6,400 to Grippotyphosa and at 1:200 to Icterohaemorrhagiae. On August 7, treatment with doxycycline was initiated at 16.5 mg twice per day for 14 days with suspected leptospirosis infection, and continued lethargy, abdominal discomfort, diarrhea, and vomiting. The symptoms resolved after the full treatment of doxycycline. On August 29, 2019, serum that was collected on June 27, 2019, prior to the red panda being relocated, was also tested on the MAT for *Leptospira* titers. Results showed positive titers of 1:25,600 and 1:100 (serogroups Grippotyphosa and Icterohaemorrhagiae, respectively).

On September 9, the red panda was again examined and noted as still mildly thin but otherwise within normal limits. Bloodwork showed increased albumin 4.2 g/dL (expected range: 2.3–4.1) and MAT results showed a titer of 1:1,600 to Grippotyphosa. Since clinical resolution does not guarantee clearance of infection and cessation of urinary shedding of leptospires (7, 9), urine samples were also collected by cystocentesis to perform fluorescent antibody testing (FAT) and culture. Two tubes each of HAN and T80/40/LH semi-solid media provided to the zoo (13, 14) were inoculated with 2–4 drops of urine on site as soon as collected. All samples were shipped overnight to the National Centers for Animal Health (NCAH) *Leptospira* working group. HAN media cultures were incubated at 37°C in 3% CO_2_, and T80/40/LH media cultures were incubated at 29°C (13, 14). FAT was performed as previously described (15). The FAT was negative; however, on September 18, 2019, the urine was culture positive in HAN media. Doxycycline, at 16.5 mg twice per day, was initiated on September 19 for 42 days. A single injection of 66,000 IU of Penicillin G was also given. On two subsequent MATs, a titer of 1:200 remained to Grippotyphosa. On May 26, 2020, there were MAT titers of 1:100 to Ballum, Grippotyphosa, and Bratislava. The animal did not show any further symptoms of leptospirosis infection and fully recovered.

## Diagnostic assessment

The isolate of *Leptospira* cultured from the red panda urine, designated strain RedPanda1, was genotyped and serotyped. The isolate was propagated in HAN liquid medium to a density of 2.5 × 10^8^/ml. For short read sequencing, DNA was extracted from a 10 ml culture using the Maxwell RSC PureFood Pathogen Kit (Promega Corporation, Madison, WI) per the manufacturer's instructions. Library preparation and sequencing were performed using the Nextera XT DNA Library Preparation Kit and the MiSeq Desktop Sequencer, 2 × 250 paired-end chemistry (Illumina, San Diego, CA) as per manufacturer's instructions. For long read sequencing, DNA was extracted from a 10 ml culture using the Circulomics Nanobind CBB Big DNA Kit (Circulomics, Baltimore, MD) using the gram-negative bacteria-high molecular weight DNA extraction protocol. The extracted DNA was quantified using the Thermo Fisher Qubit™ dsDNA BR Assay Kit (Thermo Fisher Scientific, Waltham, MA) and evaluated for quality and purity using a NanoPhotometer Pearl (Implen, Inc. Westlake Village, CA). The sequencing library beads were prepared and loaded onto Flongle flow cells for sequencing using the Oxford Nanopore Rapid Sequencing Kit (SQK-RAD004) following protocol version: RSE_9046_v1_revM_14Aug2019 (Oxford Nanopore Technologies, OX4 4DQ, UK). Sequencing was performed for a period of 24 h.

Canu v. 2.0 (16) with the default parameters was used with 101 × of Oxford Nanopore reads to assemble the complete genome consisting of two circular chromosomes. The chromosomes were initially iteratively error corrected using Pilon v. 1.23 (17) (with options “—fix bases—mindepth 5—flank 1”) and 105 × Illumina MiSeq paired-end reads until no more corrections were made. The genome chromosomes were subsequently trimmed of overlapping sequence, rotated to begin at defined start genes, and again iteratively error corrected with Pilon (using the aforementioned options) and the Illumina reads until no more corrections were made. A phylogenetic tree was made using kSNP (18). The assembled genomes were compared using the maximum likelihood output.

The final completed assembly of its genome comprised two circular genomes of 4,025,345 and 351,295 bp, with a G+C content of 36%. The genome annotation was completed by the NCBI Prokaryotic Genome Annotation Pipeline (19) and accession numbers are CP085133 and CP085134 for chromosome 1 and chromosome 2, respectively, with the following features: genes (total) 3,597, CDSs (total) 3,553, genes (coding) 3,257, genes (RNA) 44, rRNAs 1, 2, 2 (5S, 16S, 23S), tRNAs 37, ncRNAs 2, and pseudo genes (total) 296.

**Figure 2:**
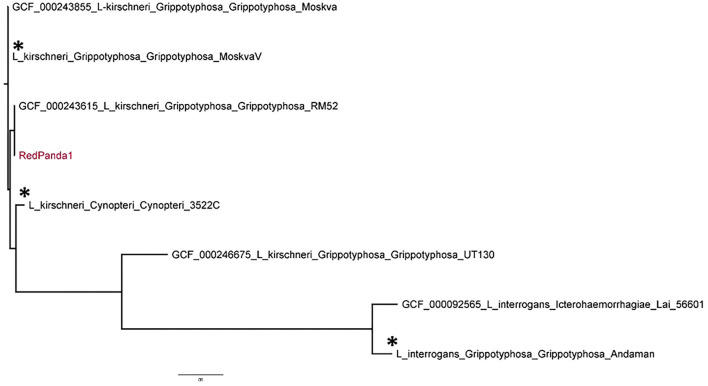
Phylogenetic tree using kSNP analysis shows strain RedPanda1 grouping with *Leptospira kirschneri* serovar Grippotyphosa and separate from *L. interrogans* isolates. For those genome sequences downloaded from GenBank, accession numbers are provided followed by species_serogroup_serovar_strain. NVSL in-house genome sequences are indicated with an asterisk (*) followed by species_serogroup_serovar_strain.

The complete genome sequence was compared to other genomes of *L. kirschneri* including strains of *L*. *kirschneri* belonging to serogroups Grippotyphosa and Cynopteri, as well as genomes of *L. interrogans* with strains of *L. interrogans* belonging to serogroup Grippotyphosa and Icterohaemorrhagiae using kSNP analysis (18) (Figure 2). The *L. interrogans* strain Lai, *L. kirschneri* strain UT130, *L. kirschneri* strain RM52 and *L*. *kirschneri* strain Moskva genome sequences were obtained from the National Center for Biotechnology Information (NCBI) database, accession numbers: GCA_000092565, GCF_000246675, GCF_000243615 and GCF_000243855 respectively. The NVSL in-house genome sequences for *L. interrogans* serogroup Grippotyphosa strain Andaman, *L. kirschneri* serogroup Grippotyphosa strain Moskva V and *L. kirschneri* serogroup Cynopteri were also included. The genome of strain RedPanda1 aligned most closely with *L. kirschneri* isolates, specifically strains belonging to serogroup Grippotyphosa serovar Grippotyphosa (Figure 2).

To determine the serogroup, the recovered isolate was sub-cultured in P-80 *Leptospira* growth media (NVSL, Ames, IA) to a density of 60–70% transmittance at 400 nm wavelength, after centrifugation to remove any auto-agglutination (600 × *g* for 15 min). The isolate was initially serotyped by MAT using *Leptospira* polyvalent reference antisera representative of 14 serogroups (Supplementary Table 1). Agglutination observed as 50% or greater was marked as positive and titrated to determine which representative serogroup had the highest titer. Once the serogroup was determined, the isolate was further serotyped to identify serovar by MAT testing with a panel of polyvalent reference antisera from serovars within that serogroup (Table 1) (20). Reference antisera determined that strain RedPanda1 belonged to the serogroup Grippotyphosa, which had a positive MAT titer of 51,200 compared to all other serogroups tested which were MAT negative. Additional serotyping with reference antisera against serovars within the serogroup Grippotyphosa predicts the isolate belongs to serovar Grippotyphosa (Table 1). Thus, the recovered isolate is typed as *L*. *kirschneri* serogroup Grippotyphosa serovar Grippotyphosa strain RedPanda1.

**Table 1:** MAT titers of the red panda isolate when tested with reference antisera against serovars within the serogroup Grippotyphosa.

**Species**	**Serogroup**	**Serovar**	**Strain**	**Titer**
*L. santarosai*	Grippotyphosa	Canalzonae	CZ 188	25,600
*L. interrogans*	Grippotyphosa	Grippotyphosa	Andaman	51,200
*L. kirschneri*	Grippotyphosa	Grippotyphosa	Moskva V	25,600
*L. kirschneri*	Grippotyphosa	Grippotyphosa	RM52	102,400
*L. interrogans*	Grippotyphosa	Muelleri	RM 2	25,600
*L. kirschneri*	Grippotyphosa	Ratnapura	Wumalasena	25,600
*L. interrogans*	Grippotyphosa	Valbuzzi	Valbuzzi	25,600
*L. kirschneri*	Grippotyphosa	Vanderhoedeni	Kipod 79	3,200

## Discussion

Pathogenic *Leptospira* comprise more than 38 pathogenic species and hundreds of serovars, and new species and serovars continue to be identified (21–24). All mammals in the zoo setting are at risk of disease since leptospires can infect such a vast number of different animal species. The zoo setting creates unique opportunities for transmission and exposure not only between exotic species, but from invasive and local wildlife. Leptospirosis is also a zoonotic disease and thus an occupational hazard in the zoo environment. Quarantine and routine testing are essential to help reduce risk of disease transmission (20). Leptospirosis in exotic animals presents a unique situation since there is little animal species-specific research on the infecting species and/or serovars of *Leptospira*, or the (sub) clinical manifestation of infection which can range from persistent urinary shedding of leptospires in asymptomatic reservoir hosts to acute fulminant infection. In this case report, a red panda was diagnosed with leptospirosis during quarantine thus enabling antimicrobial treatment to facilitate clinical resolution as well as cessation of shedding prior to cohabitating with other animals and allowing zoo staff to properly protect themselves.

Isolation and culture of pathogenic leptospires is inherently difficult due to their fastidious growth requirements and is not routinely practiced. Though some have inferred the infecting serovar based on the highest titer in an MAT reaction, this is not correct and serological results should not be confused with serovar identification. Several studies have demonstrated that inference of serovar based on patient serology is often incorrect due to paradoxical reactions, cross-reactivity, and the potential exclusion of the infecting serovar from the MAT antigen panel employed (25–27). The advent of newer media formulations has enhanced the ability to culture leptospires from animal tissues (14, 15, 28, 29). Recovery of an isolate of *Leptospira* from animal samples is definitive and essential for high resolution epidemiological typing, inclusion in antigen panels for diagnostic purposes (MAT), and to ensure bacterin-based vaccine strategies contain the relevant serovar to prevent infection and continued disease transmission.

Typing of pathogenic leptospires has evolved from serotyping to genotyping, which represent two different and unique methods for classifying leptospires that do not correlate well with each other (30). Multiple examples exist whereby the same serovar may belong to different species, e.g., serovar Hardjo may belong to either *L*. *interrogans* or *L*. *borgpetersenii*, serovar Grippotyphosa may belong to *L*. *interrogans, L*. *kirschneri* or *L*. *santarosai* (Table 1), and serovar Pomona may belong to *L*. *interrogans, L*. *kirschneri, L*. *santarosai*, or *L*. *noguchii* (31). In our case, strain RedPanda1 was genotyped as *L*. *kirschneri*, and serotyped as serovar Grippotyphosa (Table 1, Figure 2). *L*. *kirschneri* is often associated with animal and human disease, but few genomes have been sequenced. By performing complete genome sequencing, comparative genome analysis confirmed the species assignment for strain RedPanda1 which is now available for future high resolution molecular epidemiological studies and can be used to facilitate studies to further understand pathogenic mechanisms of leptospirosis.

Reservoir hosts of leptospirosis can excrete leptospires *via* urine in the absence of a detectable MAT response (32, 33). Similarly, as clinical symptoms resolve in acutely ill patients and MAT titers decline, viable leptospires may continue to be excreted in urine (9). No single assay to detect urinary shedding of leptospires is considered optimal, and the use of more than one assay to detect leptospires in urine is recommended (34). As reported here, a urine sample from the red panda post treatment was FAT negative but culture positive, which may reflect greater sensitivity of newer media formulations. In any case, culture is definitive of a live viable leptospire excreted in urine which can be both genotyped and serotyped. Live leptospires detected by culture also indicate the risk for transmission of disease to other animals and humans (35). In this case, the red panda was still shedding live leptospires, so it was important to keep her quarantined until shedding ceased.

Bacterins for leptospirosis in animals are available, but their efficacy is dependent on inclusion of relevant serovars associated with animal host infection. Since bacterins do not cross protect against multiple serovars, isolation and culture of leptospires from animal samples is essential to determine appropriate serovars for inclusion in bacterins. Recently, serovar Tarassovi was isolated from a U.S. dairy cow, but no U.S. animal vaccine includes serovar Tarassovi (28). Given the limited epidemiological data on leptospirosis in exotic animals, serovar identification can provide guidance on selection of appropriate bacterins that may be useful to manage disease outbreaks in zoos. Serovar identification can also provide guidance on the source of exposure, for example, serovar Grippotyphosa is often associated with wildlife and environmental contamination (36). In this case it was important to identify the infecting serovar so an appropriate bacterin could be used to prevent future infections in this red panda and protect the other red pandas she would be housed with.

Animals maintained in zoos and wildlife parks are at risk from infection with pathogenic leptospires from a range of sources. Though the MAT can diagnose acute leptospirosis concurrent with clinical symptoms, it cannot identify animals shedding leptospires in urine. Detection of leptospires in urine from reservoir hosts, or from patients after clinical resolution of infection, requires alternative assays such as PCR, FAT, and/or culture to accurately assess excretion and continued risk of disease to other animal and human contacts.

## Data availability statement

The datasets presented in this study can be found in online repositories. The names of the repository/repositories and accession number(s) can be found at: https://www.ncbi.nlm.nih.gov/genbank/, CP085133, CP085134.

## Ethics statement

Ethical review and approval was not required for the animal study because samples collected were part of normal diagnostic procedures.

## Author contributions

KL, KF, and JN: conceptualization. KL, KF, TA, DB, TS, JH, and JN: methodology. KL, KF, TA, DB, TS, JH, LS, and JN: formal analysis and writing—review and editing. KF, LS, and JN: resources. KL, TS, and JN: figures. KL and JN: writing—original draft preparation. All authors have read and agreed to the published version of the manuscript.
