# Supplementary material for: Isolation of Leptospira kirschneri serovar Grippotyphosa from a red panda (Ailurus fulgens) after antimicrobial therapy: Case report
Source: Front Vet Sci. 2023 Feb 2;9:1064147. doi: 10.3389/fvets.2022.1064147 (PMC9932277; doi:10.3389/fvets.2022.1064147)
Supplement: Supplementary file 1 [file Table_1.docx]

**Supplementary Table 1**: Panel of reference antisera used to identify the serogroup of the red panda isolate.

| **Species** | **Serogroup** | **Serovar** | **Strain** |
| --- | --- | --- | --- |
| *L. interrogans* | Australis | Australis | Ballico |
| *L. interrogans* | Autumnalis | Autumnalis | Akiyami A |
| *L. borgpetersenii* | Ballum | Ballum | S 102 |
| *L. interrogans* | Bataviae | Bataviae | Van Tienen |
| *L. interrogans* | Canicola | Canicola | Hond Utrecht IV |
| *L. interrogans* | Grippotyphosa | Grippotyphosa | Andaman |
| *L. interrogans* | Sejroe | Hardjo | HardjoPrajitno |
| *L. interrogans* | Icterohaemorrhagiae | Copenhageni | M20 |
| *L. interrogans* | Pomona | Pomona | Pomona |
| *L. interrogans* | Pyrogenes | Pyrogenes | Salinem |
| *L. borgpetersenii* | Tarassovi | Tarassovi | Perepelitsin |
| *L. interrogans* | Australis | Bratislava | Jez-Bratislava |
| *L. borgpetersenii* | Sejroe | Sejroe | M84 |
| *L. interrogans* | Hebdomadis | Hebdomadis | Hebdomadis |
| *L. interrogans* | Mini | Szwajizak | Szwajizak |
